# Correction: Phase I Trial of ALRN-6924, a Dual Inhibitor of MDMX and MDM2, in Patients with Solid Tumors and Lymphomas Bearing Wild-type TP53

**DOI:** 10.1158/1078-0432.CCR-21-4241

**Published:** 2022-01-19

**Authors:** Mansoor N. Saleh, Manish R. Patel, Todd M. Bauer, Sanjay Goel, Gerald S. Falchook, Geoffrey I. Shapiro, Ki Y. Chung, Jeffrey R. Infante, Robert M. Conry, Guilherme Rabinowits, David S. Hong, Judy S. Wang, Ulrich Steidl, Loren D. Walensky, Gurudatta Naik, Vincent Guerlavais, Vojislav Vukovic, D. Allen Annis, Manuel Aivado, Funda Meric-Bernstam

In the original version of this article (1), author Loren D. Walensky is mistakenly omitted from the author list. This error has been corrected in the latest online HTML and PDF versions of the article and the authors’ contributions and disclosures have been updated as necessary. The authors regret this error.

## References

[1] Saleh MN , Patel MR, Bauer TM, Goel J, Falchook GS, Shapiro GI, . Phase 1 trial of ALRN-6924, a dual inhibitor of MDMX and MDM2, in patients with solid tumors and lymphomas bearing wild-type TP53. Clin Cancer Res 2021;27:5236–47. 34301750 10.1158/1078-0432.CCR-21-0715 PMC9401461

